# Common variants in *MAEA* gene contributed the susceptibility to osteoporosis in Han Chinese postmenopausal women

**DOI:** 10.1186/s13018-020-02140-4

**Published:** 2021-01-10

**Authors:** Xuan Cai, Jun Dong, Teng Lu, Liqiang Zhi, Xijing He

**Affiliations:** 1 Department of Orthopaedic Surgery, The Second Affiliated Hospital of Xi’an Jiaotong University, No. 157 Xiwu Road, Xi’an, 710004 China; 2 Department of Joint Surgery, Honghui Hospital of Xi’an Jiaotong University, No.555, Youyi East Road, Xi’an, 710054 China

**Keywords:** Postmenopausal osteoporosis, Single nucleotide polymorphisms, Genetic association, Case-control study

## Abstract

**Background:**

Osteoporosis (OP) is a complex bone metabolism disorder characterized by the loss of bone minerals and an increased risk of bone fracture. A recent study reported the relationship of the macrophage erythroblast attacher gene (*MAEA*) with low bone mineral density in postmenopausal Japanese women. Our study aimed to investigate the association of *MAEA* with postmenopausal osteoporosis (PMOP) in Han Chinese individuals.

**Methods:**

A total of 968 unrelated postmenopausal Chinese women comprising 484 patients with PMOP and 484 controls were recruited. Four tag single nucleotide polymorphisms (SNPs) that covered the gene region of *MAEA* were chosen for genotyping. Single SNP and haplotypic association analyses were performed, and analysis of variance was conducted to test the correlation between blood MAEA protein level and genotypes of associated SNPs.

**Results:**

SNP rs6815464 was significantly associated with the risk of PMOP. The C allele of rs6815464 was strongly correlated with the decreased risk of PMOP in our study subjects (OR[95% CI]=0.75[0.63-0.89], *P*=0.0015). Significant differences in MAEA protein blood levels among genotypes of SNP rs6815464 were identified in both the PMOP (F=6.82, *P*=0.0012) and control groups (F=11.5, *P*=0.00001). The C allele was positively associated with decreased MAEA protein levels in blood.

**Conclusion:**

This case-control study on Chinese postmenopausal women suggested an association between SNP rs6815464 of *MAEA* and PMOP. Further analyses showed that genotypes of SNP rs6815464 were also associated with the blood level of MAEA protein.

**Supplementary Information:**

The online version contains supplementary material available at 10.1186/s13018-020-02140-4.

## Background

Osteoporosis (OP) is a complex bone metabolism disorder characterized by loss of bone minerals and increased risk of a bone fracture [1]. It is estimated that approximately 2% to 8% of males and 9% to 38% of females are affected in industrialized countries [2]. Early epidemiological studies indicated that the incidence of OP increases with age [3]; 15% of Caucasians in their 50s and 70% of those over 80 are affected [2]. In addition, the risk OP in women is approximately 3 times higher than in men [4, 5]. OP and fractures severely affect the quality of life of a senior individual and generate great economic and psychological burdens for the patients and their families. Imbalance between bone formation and bone resorption is the basic mechanism for all osteoporosis [6]. Multiple previous studies have indicated that OP is a complex disorder caused by both genetic and environmental factors [7–10]. Based on familial samples, the heritability of osteoporosis and bone mineral density (BMD) has been estimated to be ~50%-60% [11].

The genome-wide association study (GWAS) design has accelerated the discovery of gene-phenotype associations for complex disorders. GWAS focusing on osteoporosis and BMD have been intensively conducted in the last decade. At least 42 publications on BMD- and osteoporosis-related phenotypes have been published, and more than 200 association signals derived from approximately 90 loci that achieved genome-wide significance have been reported [12]. A large proportion of these loci are located within five major bone metabolism-related pathways, including the mesenchymal cell differentiation, WNT, NOTCH, Hedgehog and OPG-RANKRANKL signaling pathways [10]. Despite hundreds of association signals discovered by large-scale GWAS, all of these identified variants together could only explain 10–20% of the variance in bone phenotypes [10]. Therefore, more population-based studies are still needed to explore these underlying susceptible genes.

Gene macrophage erythroblast attacher (*MAEA*) is located on chromosome 4p16.3 and is expressed in a wide range of human cells, including osteoblasts and osteoclasts, which are important for bone metabolism. The encoded protein of *MAEA* is an integral membrane protein which mediates the attachment of erythroblasts to macrophages. A recent cross-sectional study suggested an association between single nucleotide polymorphism (SNP) rs6815464, a genetic polymorphism of *MAEA*, and low BMD of the total hip but not of the lumbar spine or femoral neck in Japanese women [13]. However, large-scale and well-designed replication studies in other populations are still needed to confirm this association signal.

In the present study, we aimed to investigate the association of genetic polymorphisms of *MAEA* and postmenopausal osteoporosis (PMOP) based on samples of Chinese women with PMOP and controls. To determine if serum MAEA levels associated with risk SNPs for osteoporosis, blood levels of MAEA protein were also tested.

## Methods

### Study subjects

A total of 968 postmenopausal women comprising 484 patients with PMOP and 484 controls were recruited from the Second Affiliated Hospital of Xi’an Jiaotong University (Xi’an, China) and Honghui Hospital of Xi’an Jiaotong University (Xi’an, China) from January 2014 to March 2018. For each PMOP case, control with same gender and ±3 years of age was matched. Only patients with primary osteoporosis were enrolled in the present study. Patients with secondary or idiopathic OP were not included. Individuals with a history of medication for the treatment of PMOP or medication known to affect bone metabolism within 6 months were excluded from this study. In addition, only individuals with BMI≤27 were included. To control the genetic background of our study subjects, only individuals with no migration history within the previous three generations were enrolled. Diagnosis of PMOP was made based on the BMD at the lumbar spine (L2-4) and femoral neck. Dual-energy X-ray absorptiometry (Lunar Expert 1313, Lunar Corp., USA) was used to estimate the BMD. Participants who had a T score < −2.5 SD were classified as OP. This study was approved by the Medical Ethics Committee of Xi’an Jiaotong University Health Science Center. Written informed consent was provided by all study subjects.

### SNP selection and genotyping

To maximize the genetic information coverage, we selected tag SNPs for genotyping. We established the following selection criteria: (i) the selection of SNPs located within up/downstream 10 kb of *MAEA* with minor allele frequency (MAF) greater than 0.05; (ii) the selection of tag SNPs using r2≥0.6 as the criterion. Chinese Han Beijing data in the 1000 Genomes database were utilized as a reference panel. Finally, 4 intronic SNPs were chosen for genotyping (Supplemental Table S1). Genomic DNA of our study subjects was extracted from peripheral blood leukocytes using a DNA extraction kit (Tiangen Biotech Co. Ltd, Beijing, China) according to the protocol provided by the manufacturer. SNP genotyping was performed based on the Sequenom MassARRAY platform (Sequenom, San Diego, CA, USA) according to the manufacturer’s protocol. Genotyping technicians were blinded to the labels of cases and controls during the experimental process. Fifty study subjects were randomly chosen for replication to evaluate the quality of genotyping experiments, and a 100% concordance rate was achieved. Serum MAEA levels were measured using enzyme-linked immunosorbent assay (ELISA) kits (Westtang Biotech Inc., Shanghai, China) according to the manufacturer’s protocol.

### Statistical analysis

Student’s *T* tests were performed to compare demographic and characteristic variables between patients with PMOP and controls. Hardy-Weinberg equilibrium (HWE) tests were conducted for all 4 genotyped SNPs in the control group. Genetic association analyses were performed at both genotypic and allelic levels. χ2 tests were conducted, and odds ratios with 95% confidence intervals were calculated for each SNP. In addition to the single marker-based association analysis, we also constructed the linkage disequilibrium (LD) structure and examined the genetic association at the haplotype level. To investigate whether the genetic association signal obtained at the DNA level could be reflected at the protein level, we also analyzed the relationship between blood MAEA protein level and genotypes of significant SNPs. Analysis of variance (ANOVA) was used to test the statistical significance. Bonferroni correction was applied to address multiple comparisons. For single marker-based association analyses, the threshold of *P*-values was 0.05/4=0.0125. Haploview was utilized for LD structure construction [14]. Plink was used for HWE tests and genetic association analysis [15]. Statistical computing software R was utilized for other conventional statistical analyses [16].

### Bioinformatics analysis

To examine the potential effects of significant SNPs on the gene expression of *MAEA* in multiple types of human tissues, we conducted expression quantitative trait loci (eQTL) analysis based on data extracted from the Genotype-Tissue Expression (GTEx) database [17]. In addition, we also investigated other potential candidate genes by exploring the protein-protein interaction (PPI) network of MAEA using the STRING database [18].

## Results

We examined the distributions of age, years to menopause, BMI and MAEA blood level between patients with PMOP and controls. The average blood level of MAEA protein in patients with PMOP was significantly lower than that in controls (*T*=-3.62, *P*=0.0003, Table 1). No significant differences were identified for age, BMI or years to menopause between patients with PMOP and controls.

**Table 1:** Characteristic information of the study subjects

Variables, mean±sd	Cases (N=484)	Controls (N=484)	*T*-Statistics	*P*-Values
Age, years	62.8±6.9	62.8±5.7	-0.10	0.9226
Years to Menopause, years	10.9±5.5	11.1±5.4	-0.62	0.5361
BMI, kg/m^2^	23.2±1.6	23.2±1.8	-0.28	0.7819
MAEA level, pg/ml	915.9±263.1	971.4±210.4	-3.62	0.0003

All selected SNPs were in HWE in the control group (Supplemental Table S1). SNP rs6815464 was significantly associated with the risk of PMOP (Table 1). The C allele of rs6815464 was significantly associated with a decreased risk of PMOP in our study subjects (OR [95% CI]=0.75 [0.63-0.89], *P*=0.0015, Table 2). Dosage dependence patterns were identified through genotypic analyses. The OR for the CC genotype was 0.54, and for the CT genotype it was 0.75. In addition to significant signals identified in single marker-based association analyses, we also identified haplotypic association signals. One 2-SNP LD block was obtained (rs12641735-rs6815464, Supplemental Figure S1), and further association analysis showed that this haplotype was significantly associated with PMOP (*P*=0.0057, Table 3).

**Table 2:** Results of the single marker based association analyses

CHR	SNP	POS	Genotypic Analyses						Allelic Analyses					
			Genotypes	Cases	Controls	OR[95%CI]	χ^2^	*P*-Values	Alleles	Cases	Controls	OR[95%CI]	χ^2^	*P*-Values
4	rs12641735	1310646	GG	34	24	1.57[0.91-2.71]								
			CG	184	166	1.49[0.94-1.60]			G	252	214	1.24[1.00-1.53]		
			CC	266	294	ref	4.05	0.1320	C	716	754	ref	4.08	0.0434
**4**	**rs6815464**	**1316113**	**CC**	**92**	**127**	**0.54[0.37-0.78]**								
			**CG**	**254**	**254**	**0.75[0.55-1.02]**			**C**	**438**	**508**	**0.75[0.63-0.89]**		
			**GG**	**138**	**103**	**ref**	**10.68**	**0.0048**	**G**	**530**	**460**	**ref**	**10.13**	**0.0015**
4	rs72501966	1316879	TT	13	7	2.02[0.79-5.12]								
			CT	155	134	1.26[0.95-1.66]			T	181	148	1.27[1.00-1.62]		
			CC	316	343	ref	4.43	0.1090	C	787	820	ref	3.99	0.0458
4	rs10025665	1318479	GG	58	60	0.94[0.63-1.42]								
			AG	213	216	0.96[0.74-1.26]			G	329	336	0.97[0.80-1.17]		
			AA	213	208	ref	0.11	0.9445	A	639	632	ref	0.11	0.7376

*CHR* Chromosome, *POS* Position. Threshold of *P*-Values=0.05/4=0.0125. Significant results were highlighted in bold

**Table 3:** Results of haplotype based association analyses

LOCUS	HAPLOTYPE	F_A	F_U	χ^2^	DF	*P*-Values	SNPs
H1	OMNIBUS	-	-	10.33	2	0.0057	rs12641735|rs6815464
H1	CC	0.45	0.52	10.18	1	0.0014	rs12641735|rs6815464
H1	GG	0.26	0.22	4.19	1	0.0408	rs12641735|rs6815464
H1	CG	0.30	0.26	2.62	1	0.1053	rs12641735|rs6815464

F_A: haplotype frequency in cases; F_U: haplotype frequency in controls; DF: degree of freedom

**Fig. 1:**
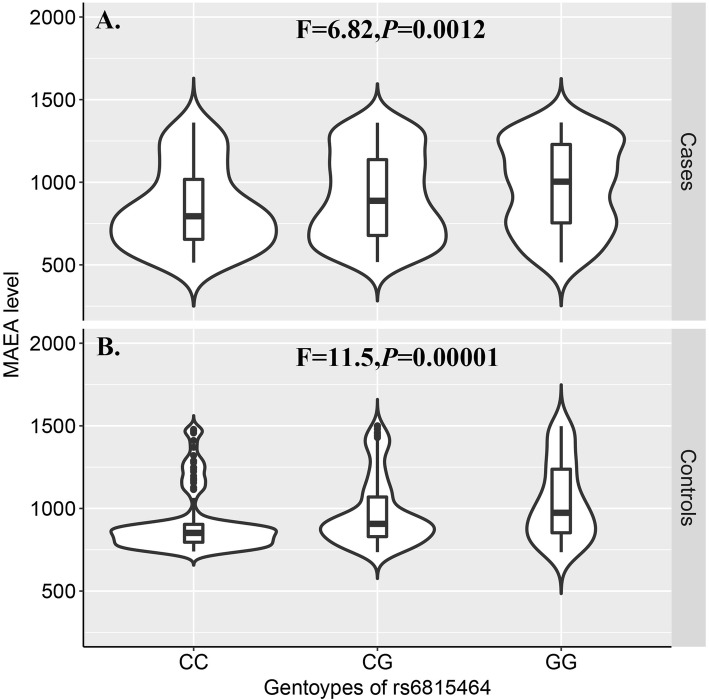
Violin and boxplots for MAEA protein level in blood in the study subjects stratified by genotypes of SNP rs6815464. A. Cases. B. Controls. *F*-statistics and *P*-Values were indicated

We further examined the differences in MAEA protein blood levels among genotypes of SNP rs6815464. Significant differences were identified in both the PMOP (F=6.82, *P*=0.0012) and control groups (F=11.5, *P*=0.00001) (Figure 1). As shown in Figure 1, the C allele was associated with decreased MAEA protein levels in peripheral blood.

**Fig. 2:**
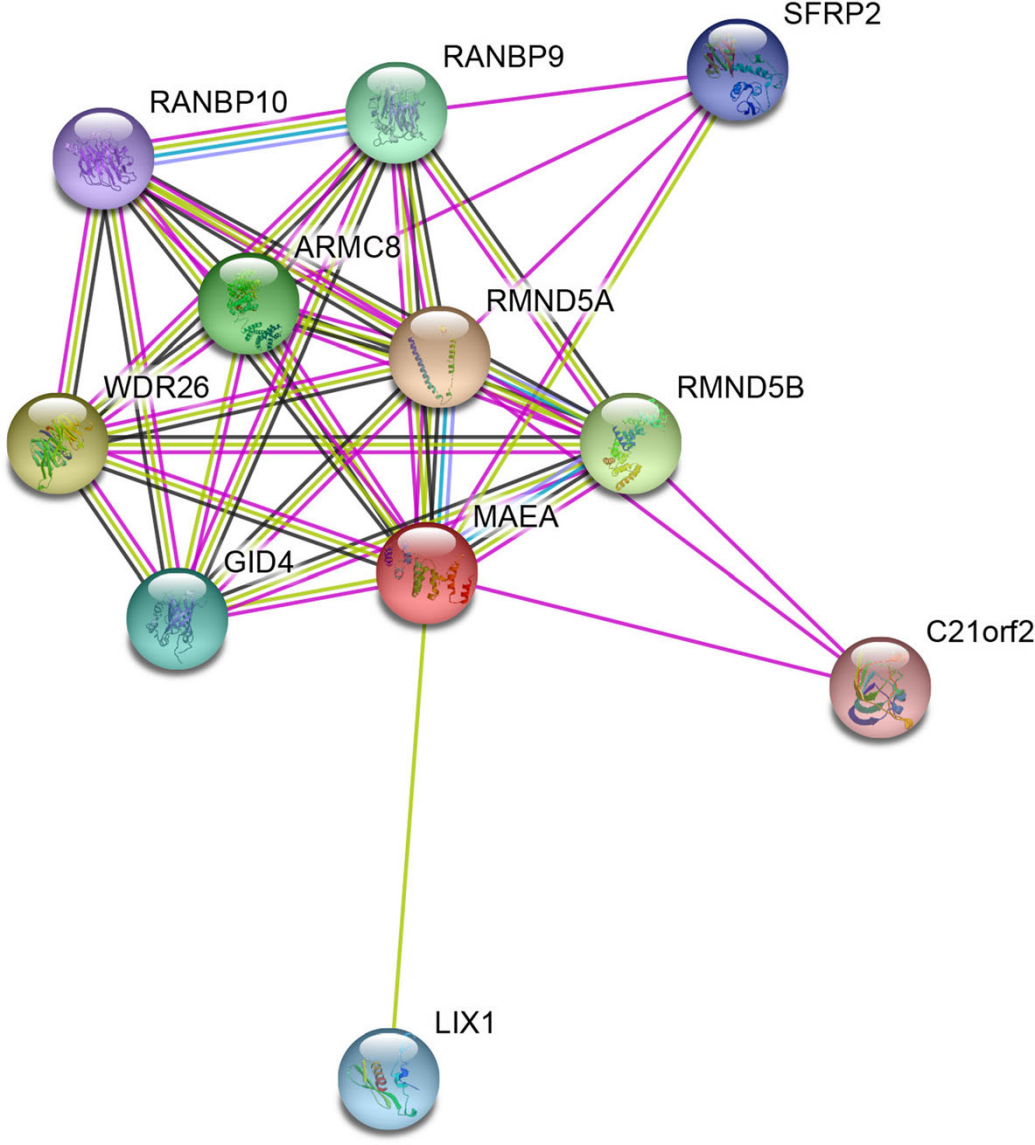
Protein-protein interactions network for MAEA and its related proteins

Genome-wide significant eQTL signals were identified for SNP rs6815464 on *MAEA* in four types of human tissues, including whole blood (*P*=1.70×10-6), sun-exposed skin (*P*=1.10×10-5), tibial nerve (*P*=2.80×10-5) and thyroid (*P*=5.00×10-5). The directions of effect were the same in all four human tissues. The C allele of SNP rs6815464 was significantly associated with decreased *MAEA* gene expression (Table 4). A PPI network of *MAEA* was constructed, and several closely connected genes, including *GID4*, *LIX1*, *RMND5A*, *RMND5B*, *ARMC8* and *WDR26,* were identified (Figure 2).

**Table 4:** cis-eQTL signals of SNP rs6815464 that achieved genome-wide significance

SNP	Gene	Ref_Allele	Alt_Allele	*P*-Values	NES	Tissue
rs6815464	*MAEA*	C	G	1.70×10^-6^	-0.17	Whole Blood
rs6815464	*MAEA*	C	G	1.10×10^-5^	-0.31	Skin - Sun Exposed (Lower leg)
rs6815464	*MAEA*	C	G	2.80×10^-5^	-0.47	Nerve - Tibial
rs6815464	*MAEA*	C	G	5.00×10^-5^	-0.44	Thyroid
rs6815464	*AC139887.4*	C	G	6.80×10^-5^	1.10	Brain - Cerebellum
rs6815464	*CTBP1*	C	G	7.80×10^-5^	0.25	Lung
rs6815464	*CTBP1-AS2*	C	G	1.10×10^-5^	-0.34	Nerve - Tibial

*NES* Normalized effect size.

## Discussion

Our results suggest that genetic polymorphisms of the *MAEA* gene could contribute to the risk of PMOP in Chinese Han populations. In a cross-sectional study performed based on the Japanese population, Yulan *et al.* reported that the G allele carriers of SNP rs6815464 were significantly associated with low BMD of the hip but not of the lumbar spine or femoral neck [13]. On the other hand, in our study, the diagnosis of PMOP was based on the BMD of the lumbar spine or femoral neck. In this sense, our findings replicated and supplemented the results of a previous study based on Japanese populations. Despite the differences in sample size, sample ethnic ancestry, study design and the targeted phenotype between our study and the previous cross-sectional study, the direction of the effect direction of SNP rs6815464 is the same, and the effect size is quite similar. SNP rs6815464 located at the intronic region of gene *MAEA* with unclear functional consequence. Therefore in addition to simple genetic association analyses at the DNA level, in the present study, we moved further to investigate the association between genotypes of SNP rs6815464 and *MAEA* gene expression levels in several types of human tissues and blood levels of MAEA protein by integrating multiple lines of evidence.

Our findings in distributions of MAEA protein blood level indicated that the genotypes of SNP rs6815464 were significantly associated with the MAEA protein level in human blood in patients with PMOP and controls. With more copies of the C allele present, the individuals have lower MAEA protein levels on average in their blood samples. This finding could, at least partly, be confirmed by our eQTL data extracted from the GTEx database. In human tissue of whole blood, the C allele was significantly associated with a decreased level of *MAEA* gene expression. It seems that the effect direction of SNP rs6815464 was the same at both the RNA and protein levels. Nevertheless, we need to be careful in interpreting these results, especially for those eQTL signals obtained from publicly available databases. The sources of the samples tested in the GTEx database were unknown, and therefore, we cannot confirm whether they are patients with OP. Since the patterns of gene expression could be very different in patients with PMOP and healthy people, the eQTL signals reported in the GTEx database might not be applicable to the present study.

Gene *MAEA* encodes an integral membrane protein that mediates the attachment of erythroblasts to macrophages [19]. A recent study indicated that deletion of Maea in mouse macrophages caused severe reductions in bone marrow macrophages, erythroblasts, and in vivo island formation [19]. MAEA has an important role in hematopoiesis in BM, including granulocyte-macrophage colony-forming units, which are the monocytes of osteoclasts [20]. In addition, a recent study also indicated that genetic polymorphisms of *MAEA* contributed to the risk of periodontitis [21]. In the present study, the C allele of SNP rs6815464, which was associated with a decreased risk of PMOP, was associated with both lower RNA and protein levels in human blood. Based on these results, we hypothesized that MAEA might be involved in the genesis of osteoclasts. A reduction in MAEA expression might cause a decrease in BM macrophages and have a protective effect on PMOP through blocking the genesis of osteoclasts. Further functional analyses based on animal models are still needed to unravel the pathological mechanisms of MAEA protein and PMOP.

Despite the evidence that SNP rs6815464 is associated with gene expression level of *MAEA* and blood level of MAEA protein, it is too early to confirm that this SNP is the DNA variant with true biological effect. It is likely that SNP rs6815464 is just a surrogate of some other underlying DNA variants. These underlying DNA variants could be some other genetic polymorphisms that were not genotyped in the present study, or they could be some low-frequency or rare DNA variants that were not considered in the current study. Multiple lines of evidence have shown that these low-frequency and rare DNA variants might play an important role in the onset and development of some complex disorders, including osteoporosis [22–25]. The genotyping technology and SNP selection strategy utilized in the present study cannot capture the information of these low-frequency DNA variants. Therefore, in the future, next-generation sequencing-based studies should be conducted to thoroughly investigate the genetic architecture of *MAEA* and its effects on PMOP.

This study has several limitations. Only postmenopausal women were included as study subjects, although OP is a common disease in both senior men and women. This strategy accelerated the sample recruitment process because the incidence of OP in postmenopausal women is much higher than that in senior men. However, on the other hand, this strategy makes it more difficult to generalize our results to wider populations. Another limitation is that we did not perform any statistical procedures to adjust the potential population stratification. Population stratification is a potential confounder for genetic association mapping. As a candidate gene based study with only 4 SNPs genotyped, it is very difficult for us to adjust this confounder using some standard procedures such as principle component analysis or genomic control. Nevertheless, in the sample recruitment process, we restricted our study subjects by their immigration history to control their genetic backgrounds. We believe that this procedure would, at least partly, control the potential population stratification.

In summary, this case-control study based on Chinese postmenopausal women indicated an association signal between the GG genotype of rs6815464 in *MAEA* gene and the increased risk of PMOP. Further analyses showed that genotypes of SNP rs6815464 were also associated with the blood level of MAEA protein. These results suggested that rs6815464 in *MAEA* gene may be genetic factors affecting the risk of osteoporosis in Chinese postmenopausal women.

## Supplementary Information

**Additional file 1: Supplemental Tables S1.** Genetic information of the 4 genotyped SNPs. **Supplemental Figure S1.** Linkage disequilibrium structure of the 4 genotyped SNPs. Values of D’ were indicated in each cell. LD block was indicated by black bold frame.

## Data Availability

Please contact the authors for reasonable requests.

## Abbreviations

OP: Osteoporosis
BMD: Bone mineral density
GWAS: Genome-wide association study
MAEA: Macrophage erythroblast attacher
SNP: Single nucleotide polymorphism
PMOP: Postmenopausal osteoporosis
MAF: Minor allele frequency
ELISA: Enzyme-linked immunosorbent assay
HWE: Hardy-Weinberg equilibrium
LD: Linkage disequilibrium
ANOVA: Analysis of variance
eQTL: Expression quantitative trait loci
PPI: Protein-protein interaction
GTEx: Genotype-tissue expression
ORs: Odds ratios
