# Supplementary material for: Common variants in MAEA gene contributed the susceptibility to osteoporosis in Han Chinese postmenopausal women
Source: J Orthop Surg Res. 2021 Jan 10;16:38. doi: 10.1186/s13018-020-02140-4 (PMC7798333; doi:10.1186/s13018-020-02140-4)
Supplement: Supplementary file 1 — Additional file 1: Supplemental Tables S1. Genetic information of the 4 genotyped SNPs. Supplemental Figure S1. Linkage disequilibrium structure of the 4 genotyped SNPs. Values of D’ were indicated in each cell. LD block was indicated by black bold frame. [file 13018_2020_2140_MOESM1_ESM.docx]

**Supplemental Tables S1**. Genetic information of the 4 genotyped SNPs.

| CHR | POS | SNP | A1 | A2 | MAF | HWE | FUNC |
| --- | --- | --- | --- | --- | --- | --- | --- |
| 4 | 1310646 | rs12641735 | G | C | 0.24 | 0.90 | intron |
| 4 | 1316113 | rs6815464 | C | G | 0.49 | 0.27 | intron |
| 4 | 1316879 | rs72501966 | T | C | 0.17 | 0.16 | intron |
| 4 | 1318479 | rs10025665 | G | A | 0.34 | 0.76 | intron |

CHR: chromosome; POS: position; A1: minor allele; A2: major allele; MAF: minor allele frequency; HWE: *P*-Values for Hardy-Weinberg equilibrium tests conducted in controls; FUNC: functional region.


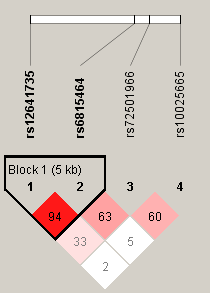


**Supplemental Figure S1.** Linkage disequilibrium structure of the 4 genotyped SNPs. Values of D’ were indicated in each cell. LD block was indicated by black bold frame.
